# Ectodermal Influx and Cell Hypertrophy Provide Early Growth for All Murine Mammary Rudiments, and Are Differentially Regulated among Them by Gli3

**DOI:** 10.1371/journal.pone.0026242

**Published:** 2011-10-27

**Authors:** May Yin Lee, Victor Racine, Peter Jagadpramana, Li Sun, Weimiao Yu, Tiehua Du, Bradley Spencer-Dene, Nicole Rubin, Lendy Le, Delphine Ndiaye, Saverio Bellusci, Klaus Kratochwil, Jacqueline M. Veltmaat

**Affiliations:** 1 Institute of Molecular and Cell Biology, A*STAR (Agency for Science, Technology and Research), Singapore, Singapore; 2 Graduate School for Integrative Sciences and Engineering, National University of Singapore, Centre for Life Sciences (CeLS), Singapore, Singapore; 3 Bio-Informatics Institute, A*STAR (Agency for Science, Technology and Research), Singapore, Singapore; 4 Experimental Histopathology Laboratory, Cancer Research UK London Research Institute, London, United Kingdom; 5 Developmental Biology Program, The Saban Research Institute of Children's Hospital Los Angeles, Los Angeles, California, United States of America; 6 Institut Curie/CNRS-UMR144, Equipe de Morphogenèse Cellulaire et Progression Tumorale, Paris, France; 7 Molecular Biology Institute, Salzburg, Austria; 8 Department of Anatomy, Yong Loo Lin School of Medicine, National University Health System, Singapore, Singapore; Baylor College of Medicine, United States of America

## Abstract

Mammary gland development starts *in utero* with one or several pairs of mammary rudiments (MRs) budding from the surface ectodermal component of the mammalian embryonic skin. Mice develop five pairs, numbered MR1 to MR5 from pectoral to inguinal position. We have previously shown that *Gli3^Xt-J/Xt-J^* mutant embryos, which lack the transcription factor Gli3, do not form MR3 and MR5. We show here that two days after the MRs emerge, *Gli3^Xt-J/Xt-J^* MR1 is 20% smaller, and *Gli3^Xt-J/Xt-J^* MR2 and MR4 are 50% smaller than their wild type (wt) counterparts. Moreover, while wt MRs sink into the underlying dermis, *Gli3^Xt-J/Xt-J^* MR4 and MR2 protrude outwardly, to different extents. To understand why each of these five pairs of functionally identical organs has its own, distinct response to the absence of Gli3, we determined which cellular mechanisms regulate growth of the individual MRs, and whether and how Gli3 regulates these mechanisms. We found a 5.5 to 10.7-fold lower cell proliferation rate in wt MRs compared to their adjacent surface ectoderm, indicating that MRs do not emerge or grow via locally enhanced cell proliferation. Cell-tracing experiments showed that surface ectodermal cells are recruited toward the positions where MRs emerge, and contribute to MR growth during at least two days. During the second day of MR development, peripheral cells within the MRs undergo hypertrophy, which also contributes to MR growth. Limited apoptotic cell death counterbalances MR growth. The relative contribution of each of these processes varies among the five MRs. Furthermore, each of these processes is impaired in the absence of Gli3, but to different extents in each MR. This differential involvement of Gli3 explains the variation in phenotype among *Gli3^Xt-J/Xt-J^* MRs, and may help to understand the variation in numbers and positions of mammary glands among mammals.

## Introduction

In mouse embryos, five pairs of mammary rudiments (MRs) arise asynchronously between embryonic day (E) 11 and E12, along a pair of histologically and molecularly distinct ‘mammary’ lines (ML) of ectoderm; one line on each flank, extending between axilla and inguen along the ventro-lateral boundary of the flank [1]. Initially disk-shaped multilayered placodes, MRs grow rapidly and become bud- or bulb-shaped within 2 days [2]. While the ectodermal origin of MRs has been demonstrated [3], the cellular mechanisms orchestrating the formation and early growth of MRs remain ill-understood.

The few studies focusing on unraveling these mechanisms leave caveats. For example, Balinsky tested whether murine MRs grow by enhanced cell proliferation. Due to technical limitations of his time, he had to pool MRs - all or a subset, that's unclear - from E11 to E14 embryos for statistical analysis, and found a significant 3.5-fold lower fraction of mitotic cells within the MRs compared to pooled ectoderm and epidermis [4]. He therefore suggested that MRs do not grow by cell proliferation, but by recruitment of ectodermal cells, most likely via centripetal aggregation [5]. However, he neither demonstrated ectodermal recruitment, nor investigated whether the lower mitotic index of MRs simply reflected the negatively allometric growth of MRs with the embryo that he had also found [4]. In rabbit, MRs were later shown to recruit ectodermal cells, as charcoal distributed on but not adjacent to the ridge-like ML of E13 rabbit embryos, is incorporated into the emerging MRs over a period of 24–48 hours [6]. Propper therefore concluded that ectodermal cells migrate along the mammary ridge to accumulate into the MRs. Contrary to the concept of centripetal aggregation, Propper proposed cell migration along the length of the mammary ridge, by attributing migratory properties to superficial spindle-like cells aligning with the length of the mammary ridge of fixed rabbit embryos [7].

Following the molecular identification of a ML in the mouse embryo [1], Propper's concept of cell migration along the ML as a mechanism of MR growth was extrapolated to the mouse embryo, by comparing TOPGAL-expressing cells along the surface of the murine ML to the spindle-like cells on the rabbit's mammary ridge [8]. However, such extrapolation may not be justified, because of several differences in early mammogenesis between mouse and rabbit. For example, the murine ML is much thinner than the rabbit's mammary ridge; it becomes histologically and molecularly distinct almost simultaneously with, instead of prior to the appearance of the MRs as occurs in rabbit; the murine MRs appear as elevated domes along the ML while in rabbit, MRs are left behind as residual peaks following subsidence of the mammary ridge; and the murine ML disappears relatively early compared to the developmental stage of the MRs [1], [5], [6], [9]. Thus, in mouse, the ML may be unable to provide sufficient cells to account for MR growth. Yet to what extent ectodermal recruitment does contribute to murine MR growth, and whether alternative cellular mechanisms of growth are involved, has not been explored.

We previously suggested that different molecular requirements for mammary induction may exist along the ML [2]. This suggestion is now supported by the regional instead of global effects along the ML of at least 10 mutated genes [9], [10], [11], [12], [13], [14], [15]. For example, similar to *Xt*-mutants [16], *Gli3^Xt-J/Xt-J^* null mutants of the transcription factor Gli3 [17] fail to induce MR3 and 5, but not MR1, 2 and 4 [9], [18], [19]. Because such different molecular mechanisms of the individual MRs may culminate into different cellular mechanisms driving MR induction and early growth, each MR should be examined individually to fully understand mammary development.

Here we report growth defects in *Gli3^Xt-J/Xt-J^* MR1, MR2 and MR4, coinciding with the daily switching morphogenetic stages. To determine the cause of stunted growth of *Gli3^Xt-J/Xt-J^* MR1, MR2 and MR4, we considered that growth may be mediated by cell proliferation, hypertrophy, cell survival, recruitment from adjacent ectoderm, or a combination of these events. As these mechanisms may vary per MR and morphogenetic stage, we examined all MR pairs as separate entities and at discrete days. We developed image analysis algorithms to quantify the early growth rates and proliferation rate by BrdU-incorporation, and to generate 3D-reconstructions of the individual MRs. We found ≤4.5% BrdU-incorporation within 2 hours in the growing MRs, indicating that proliferative activity is too low to explain MR growth. While we faced experimental obstacles to assay cell migration via time-lapse video-analysis, this low BrdU-incorporation enabled us to use incorporated BrdU as a cell tracer at 24 hours after labeling. We detected that ectodermal cells, recruited mostly if not all from *outside* the ML, contribute significantly to growth of all MRs during the first day of mammary development. Cell hypertrophy is a major growth contributor during the second day. We identified a role for Gli3 in cell proliferation, survival, migration, and hypertrophy, and differences therein among the MRs themselves and in their adjacent tissues. Such rudiment-specific molecular regulation of shared cellular mechanisms provides a beginning to the understanding of patterning and early growth of MRs within the ectoderm, and may have to be taken into consideration in studies of postnatal mammary gland development and tumorigenesis.

## Materials and Methods

### Mice and Ethics Statement

*Gli3^Xt-J/+^* mice on a C57BL/6J background (Jackson Laboratories, stock 000026) were bred and kept in strict accordance with the recommendations in the Guide for the Care and Use of Laboratory Animals of the National Institutes of Health. Steps were taken to minimize suffering. The research protocol was approved by the Institutional Animal Care and Use Committee of Childrens Hospital Los Angeles (permit 29-02 to SB) and A*STAR Biological Resource Centre (permits 060204 and 090463 to JMV). *Gli3^Xt-J/Xt-J^* embryos from timed *Gli3^Xt-J/+^* intercrosses, with noon of the day of vaginal plug appearance considered as E0.5, were genotyped as described [17].

### Section *in situ* hybridization

4% PFA-fixed embryos were paraffin embedded ( = PFPE) and transversally sectioned at 8 µm thickness for *in situ* hybridization with a ^35^S-labeled *Gli3* antisense probe as described [20].

### Micro-array analysis

Embryos were harvested in RNA-later (Ambion). Ectoderm and mammary epithelium were separated from the mesenchyme by microsurgical techniques (Sun *et al.*, in preparation). Minimal tissue cross-contamination was confirmed by analysis of tissue-specific transcript expression. We generated five E12.5 and two E13.5 independent pools of the five mammary rudiment (MR) pairs, ectoderm and mesenchyme respectively of at least five embryos. Per pool, 10 ng of high-quality RNA, extracted with the RNeasy Micro Plus Kit (Qiagen), was amplified with WT-Ovation™ Pico System to produce labeled cDNA (NuGene Technologies, Inc.) for hybridization to Affymetrix Mouse Gene 1.0 ST micro-array chips. Data were analyzed with the Partek Genomics Suite.

### Immunohistochemical detection of BrdU incorporation

Day 11.5 to 13.5 pregnant mice were injected intraperitoneally with 1 ml/100 g bodyweight BrdU (Amersham). Embryos were harvested at 2 or 24 hours post-injection, methanol-Carnoy's-fixed (60%MeOH/30%HCl_3_/10%HAc), paraffin embedded ( = MCFPE),and transversally sectioned at 5–6 µm thickness. BrdU-incorporation was detected with anti-BrdU (1∶100, Amersham), anti-mouse HRP (1∶500, Jackson), and the substrate diaminobenzidine. Substrate conversion was stopped at saturation, but before background signal developed. Slides were counter-stained with Harris hematoxylin (Sigma-Aldrich). Figure panels show the central section of representative MRs.

### Apoptotic cell detection

TUNEL assays were performed on 5 or 6 µm sections of PFPE or MCFPE embryos with the *in situ* cell death detection kit (Roche). Figure panels show the central section of representative MRs.

### Quantitation of BrdU incorporation

We developed algorithms in Matlab R2008b (Mathworks) to quantify BrdU-labeling in digital images loaded into Metamorph 7.5.1 (Molecular Devices Corp.). First, digital images were segmented with manually drawn contours along the basement membrane of the ectoderm; around the MR and its neck if present. An automatic active contour [21], [22] further segmented the ectoderm from the background. The MR-contour was automatically congruently dilated, and in the central sections also eroded [23] with empirically determined parameters: A 15 µm dilation included the few layers of mammary mesenchyme that differentiate from the surrounding dermal mesenchyme by condensation and Androgen Receptor expression between E12.5 and E13.5 [24]; and erosion to yield a 1∶3 area ratio coincides with histological and immuno-chemical differences between cuboidal core cells and larger columnar peripheral cells at bud-stage. A manually drawn line segmented the mammary mesenchyme of *Gli3^Xt-J/Xt-J^* MR2. Two automatically placed lines parallel to the ectoderm - one through the center of the MR and the other 65 µm below it - were empirically determined to include dermal mesenchyme while excluding the somites and some laterally extending mammary mesenchyme around the top half of each MR at E13.5. The algorithms returned quality control images for verification of segmentation. Manual color thresholding segmented BrdU^+ve^ nuclei and all nuclei within the images. Numerical output on total nuclear and BrdU^+ve^ areas per segment were written to an Excel file.

### Volume measurements

Using our abovementioned software, the area (in µm^2^) of mammary epithelium was quantified in consecutive sections through entire MRs of MCFPE embryos. We developed a software in C programming language on CentOS 5.3 (http://www.centos.org) to plot values against cumulative section thickness, apply Levenberg-Marquardt curve-fitting [25] to reduce errors created by lost sections, and then determine the MR volume as the area under the fitted curve.

### Statistical analysis

Unless indicated otherwise, MRs of one or both flanks of wt and *Gli3^Xt-J/Xt-J^* littermates from at least 3 different litters were used per age-group. Data are represented as mean ± standard deviation. Asterisks in bar graphs indicate a significant difference (*p<*0.05) between samples connected by a horizontal bracket, as tested with Student's *t-*test if BrdU-exposure conditions were different (*e.g.* comparing 2 h and 24 h post labeling), or a paired Student's *t-*test if BrdU-exposure conditions were the same (*e.g* comparing littermates, or tissues within specimens). Significance of differences among all MRs was tested with ANOVA.

### 3D-reconstruction of the MRs

We manually segmented (labeled) the ectoderm and MR in each image using segmentation editor in Fiji (http://pacific.mpi-cbg.de/wiki/index.php/Fiji). Algorithms were developed in Matlab R2008b and R2010b (Mathworks) to determine two transformation control points in the ectoderm in addition to the MR-center. These three points were used to align consecutive images with the first image of the stack via non-reflective similarity transformation [26]. The aligned original image-stacks were converted to inverted grayscale from 0 to 1.0, after which the black (BrdU^+ve^) pixels were segmented from the background using an empirically determined fixed threshold value of 0.65. With *à priori* knowledge of the approximate nuclear size, we eliminated noise or artifacts. Touching nuclei were split using the Evolving Generalized Voronoi Diagram algorithm [27]. Aligned labeled images and segmented original images served to generate 3D iso-surfaces of ectoderm, MR, and BrdU^+ve^ nuclei, in different superimposed transparent colors.

## Results

### Each Gli3^Xt-J/Xt-J^ mammary rudiment pair has a unique phenotype

**Figure 1:**
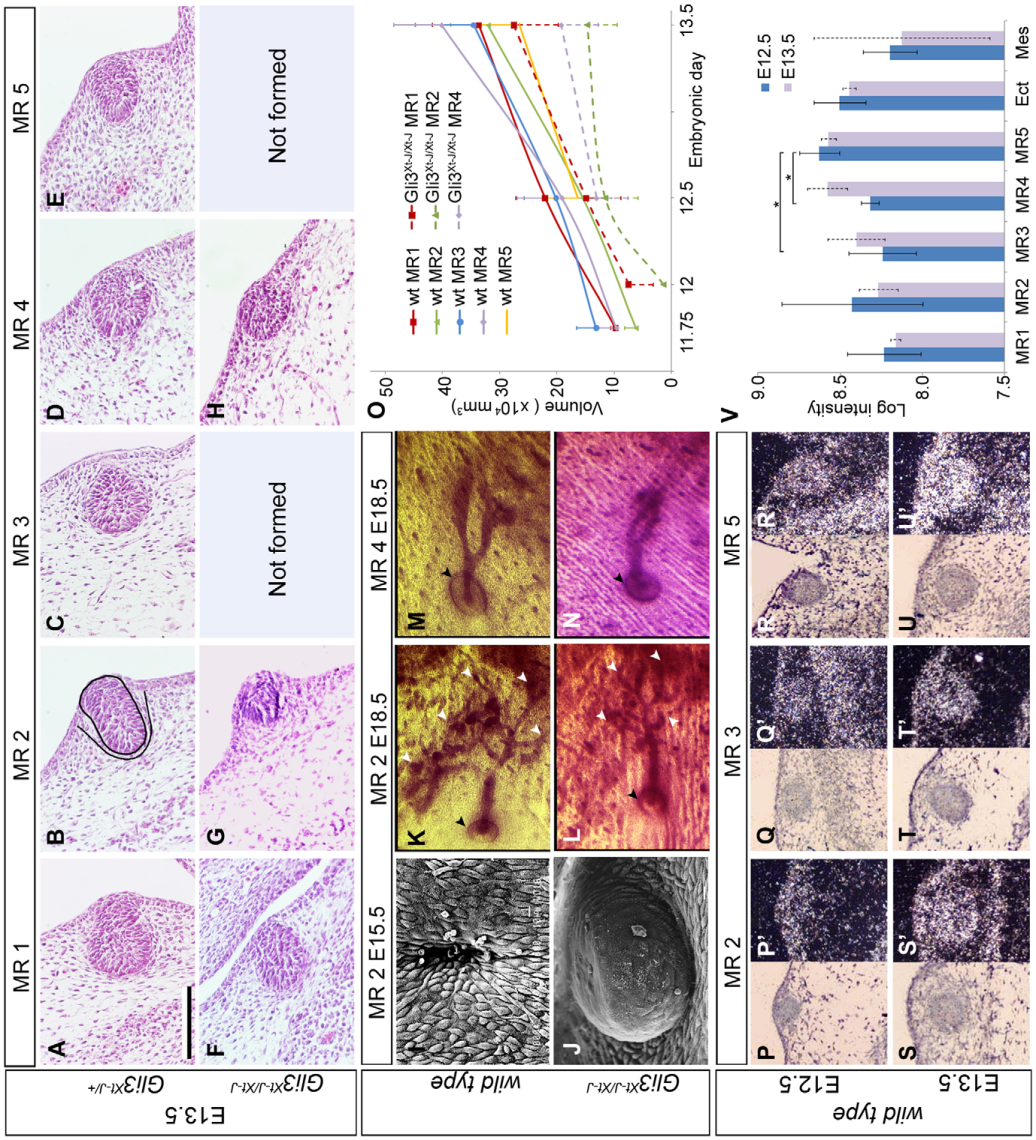
Relationship between Gli3 expression and mammary rudiment growth in mouse embryos. **A–H:** Hematoxylin/Eosin stained central transversal sections through MRs. Black contours in B surround the MR (solid) and mammary mesenchyme (dotted). Scale-bar in A: 100 µm in A–H. **I–J:** Scanning electron micrographs showing the outlet of the prospective milk-canal in a wt MR2, and the outwardly protruding *Gli3^Xt-J/Xt-J^* MR2 at E15.5. **K–N:** Carmine-stained skins. Arrowheads indicate some end buds (white) and the nipple (black). **O:** Volumetric growth curve of MRs. **P–U′:** Bright-field images of wt MRs and corresponding dark-field images with the radio-active *in situ* hybridization signal of a *Gli3* mRNA probe in white. **V:** Quantification of *Gli3* expression in micro-array data of each MR, ectoderm (Ect) and mesenchyme (Mes). Dashed error-bars at E13.5 extend between *n* = 2 measurements.

We previously reported that *Gli3^Xt-J/Xt-J^* mutants fail to induce MR3 and MR5 at E11.5 [9], [18]. Current analysis revealed that *Gli3^Xt-J/Xt-J^* MR1 is slightly, and MR2 and MR4 are obviously smaller than their wild type (wt) (Fig. 1A–H) or heterozygous (not shown) counterparts at E13.5. At E12.5, *Gli3^Xt-J/Xt-J^* MR2 and MR4 look like placodes instead of hillocks; and by E13.5 MR4 has a mild, and MR2 a severe invagination defect (Fig. 1 A–H, 2A–Y). Whereas MR4 does invaginate by E15.5 and forms an ectodermal indentation preceding formation of an outlet of the milk canal (not shown), MR2 continues to grow outwardly without forming an outlet (Fig. 1I,J). Nonetheless, *Gli3^Xt-J/Xt-J^* MR2 undergoes nipple formation, sprouting and branching morphogenesis before E18.5, as do MR4 (Fig. 1K–N) and MR1 (not shown). In conclusion, each of the developing *Gli3^Xt-J/Xt-J^* MRs has a distinct phenotype.

**Figure 2:**
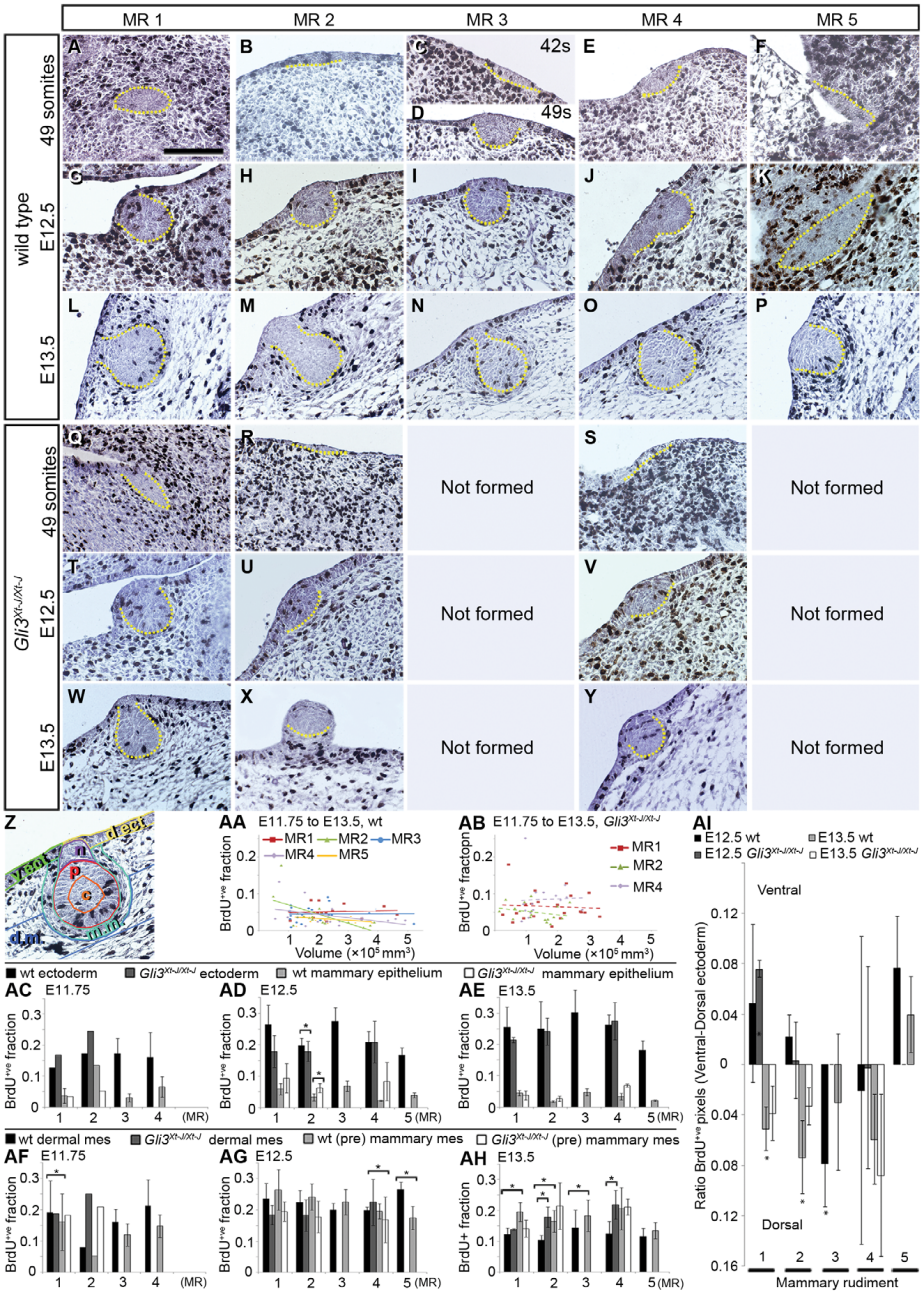
Proliferative activity in wt and *Gli3^Xt-J/Xt-J^* MRs and adjacent tissues. **A–Y:** Immunohistochemical detection of BrdU-incorporation (black) at 2 hours post-labeling, with hematoxylin-counterstain (blue). Dashed yellow lines outline the MR. Left is ventral ectoderm, in some panels indicated with *. Scale-bar in A: 100 µm in A–Y. **Z-AI:** Quantification of BrdU-incorporation in entire MRs and their adjacent tissues, represented in A–Y. **Z:** Quality control image of a wt E13.5 MR1 as generated with our software. Colored outlines demarcate dorsal ectoderm (d.ect., green), ventral ectoderm (v.ect., yellow), MR core (c, orange), MR periphery (p, red), MR neck (n, purple), mammary mesenchyme (m.m., turquoise), and dermal mesenchyme (d.m., blue). **AA,AB:** Relationship between MR-volume and BrdU-incorporation. **AC–AE:** The BrdU^+ve^ fraction of all nuclear pixels in MRs and their adjacent ectoderm. **AF–AH:** The BrdU^+ve^ fraction of all nuclear pixels in ‘pre’mammary or mammary and dermal mesenchyme (mes). **AI:** Differences in BrdU-incorporation between the ventral and dorsal ectoderm (fraction in dorsal side subtracted from fraction in ventral side per embryo). Asterisks denote a significantly difference (*p<*0.05) between both sides.

In serial histological sections, we measured MR size between E11.75 - when all wt MRs except MR5 can be accurately segmented from the ectoderm - and E13.5 - when the main growth defect has occurred. At E11.75, wt MRs vary in size between 6.2×10^4^ µm^3^ (MR2) and 13×10^4^ µm^3^ (MR3) in accordance with MR3 emerging before MR2 [1], [18]. All MRs grow at different rates (Fig. 1O), to attain a size of 2.6–4.0×10^5^ µm^3^ by E13.5 (Table 1). The developing *Gli3^Xt-J/Xt-J^* MRs are about ¼ day delayed in their onset, and MR4 is initially so flat that it can only be reliably segmented from the ectoderm from E12.5 onwards. MR1 grows normally, but does not compensate for the delayed start and remains about 20% smaller than in wt. *Gli3^Xt-J/Xt-J^* MR2 and MR4 grow slowly, such that by E13.5 they are ∼50% smaller than in wt, and even smaller than E12.5 wt MRs (Fig. 1O).

**Table 1:** Growth comparison of mammary rudiment volume to embryonic weight in wt E11.75–E13.5 mouse embryos.

	V_E11.75_ (*n*)	V_E12.5_ (*n*)	V_E13.5_ (*n*)	V_E12.5_/V_E11.75_	V_E13.5_/V_E12.5_	V_E13.5_/V_E11.75_
**MR 1**	9.8±0.8 (*4*)	22.1±5.1 (*16*)	33.6±8.1 (*13*)	2.3	1.5	3.4
**MR 2**	6.2±1.9 (*2*)	15.5±3.6 (*14*)	32.1±6.4 (*14*)	2.5	2.1	5.2
**MR 3**	13.1±3.5 (*8*)	20.2±5.5 (*14*)	34.5±10.2 (*13*)	1.5	1.7	2.6
**MR 4**	9.6±3.7 (*6*)	19.1±7.9 (*17*)	40.2±8.3 (*14*)	2.0	2.1	4.2
**MR 5**	*n.d*	16.4±5.2 (*15*)	26.5±7.4 (*14*)	*n.d*	1.6	*n.d*

	W_E11.5_ *(n)*	W_E12.5_ *(n)*	W_E13.5_ *(n)*	W_E12.5_/W_E11.5_	W_E13.5_/W_E12.5_	W_E13.5_/W_E11.5_
**Embryo**	30.6±6.3 (*6*)	82.8±13.3 (*5*)	134.6±12.9 (*14*)	2.7	1.6	4.4

Embryos were dissected in PBS, dried with filter paper, and weighed with a mimimum of adhering PBS. Abbreviations: wt: wild type; V: Volume (×10^4^ µm); MR: mammary rudiment; W: Weight (mg).

*In situ* hybridization revealed no detectable *Gli3* expression in the ectoderm or the emerging epithelium of mammary line (ML) and placodes at around E11.5, but high expression in the thoracic and lumbar somites and delaminating dermal mesenchyme [9]. Thus, the initial growth defect of the *Gli3^Xt-J/Xt-J^* MR1, MR2 and MR4 is likely due to loss of *Gli3* in these mesodermal derivatives and consequently disturbed interactions with the ectoderm. However, by E12.5 and E13.5, *Gli3* is detectable *in situ* in the (prospective) mammary mesenchyme, ectoderm, and mammary epithelium (Fig. 1P–U′). Micro-array analysis of separated tissues at these ages confirms this mRNA expression profile (Fig. 1V), which is also consistent with the Gli3 protein expression profile at E13.5 [19]. Thus, the initial growth defects of *Gli3^Xt-J/Xt-J^* MR2 and MR4 at E11.5 could subsequently be compounded by the lack of *Gli3* in any of the tissue compartments.

### Peripheral cells in Gli3^Xt-J/Xt-J^ MR2 and MR4 fail to differentiate into large columnar cells

Slightly before E12.5, peripheral cells within wt MR1, wt MR3 and *Gli3^Xt-J/Xt-J^* MR1 undergo hypertrophy and become columnar (Fig. 2G,I,T), associated with altered protein expression levels (not shown), while cells in the ectoderm and core of these MRs remain cuboidal. During the following day, hypertrophy also occurs in peripheral cells of the other wt MRs, but not of *Gli3^Xt-J/Xt-J^*MR2 and MR4 (Fig. 1A–H, 2A–Y). The hypertrophy-mediated volume increase of MRs can be estimated by considering a MR as a sphere with a realistic radius *r* of 5 cell-diameters and with the outer cell doubling its diameter in 1 day (thus *r* = 6 cell diameters). Then the sphere's volume V = 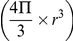 would increase 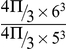
≈1.7 fold, which corresponds reasonably well with the observed growth between E12.5 and E13.5 (Table 1). Given the observed shape changes between E12.5 and E13.5 (Fig. 2), this calculation probably yields an overestimation, but indicates nonetheless that hypertrophy into columnar cells may contribute considerably to MR growth between E12.5 and E13.5. Hence, the lack of hypertrophic differentiation in *Gli3^Xt-J/Xt-J^* MR2 and MR4 by E13.5 (Fig. 1G,H, 2X,Y) may explain, at least partly, the reduced growth of these MRs between E12.5 and E13.5.

### Emergence and growth of the MRs is not mediated by enhanced cell proliferation

But what explains the smaller size of *Gli3^Xt-J/Xt-J^* MR2 and MR4 at E12.5, before hypertrophy should occur? Histological examination readily visualizes that their smaller size is mainly due to a reduced cell number, or hypoplasia (Figs. 2H,U; 2J,V). To address whether this reflects a proliferation defect, we first determined to what extent cell proliferation contributes to MR growth in wt embryos. We labeled cells in S-phase with BrdU. Transversal sections of E11.25 (or 42 somite-stage) to E13.5 embryos reveal that the ML and MRs contain strikingly few BrdU^+ve^ cells compared to their flanking ectoderm (Fig. 2A–Y). Thus, ectodermal multilayering during ML and subsequent MR formation are not a consequence of locally enhanced cell proliferation.

To quantify proliferation rates, we developed image analysis algorithms to segment images of histological sections in MRs (with neck, periphery and core), ectoderm, dermal and mammary mesenchyme, and in BrdU^+ve^ and hematoxylin^+ve^ areas. The algorithms generated quality control images displaying all segmentation boundaries, for visual inspection of segmentation and validity of the numerical output regarding relative BrdU-content of segments (Fig. 2Z).

The ratio of BrdU^+ve^ pixels among all nuclear (BrdU^+ve^+hematoxylin^+ve^) pixels serves as a proxy for proliferation rate. In embryos younger than E11.75, most MRs are too flat for accurate segmentation. In wt MRs larger than 10×10^4^ µm^3^ (Fig. 2AA), *i.e.* at E12 and older (Fig. 1O), the average proliferation rate appears relatively constant, as is the case in *Gli3^Xt-J/Xt-J^* MRs at all volumes (Fig. 2AB). We therefore excluded a need to correct for differences in developmental stage of the MR at any chronological age of embryos, and henceforth grouped the data by chronological age.

The proliferation rate within wt MRs is very low: 4.5%±2.4 at E12.5 and 3.3%±1.5% at E13.5 (Fig. 2AD, AE), and varies significantly among MRs (*p = *0.0002 at E12.5; *p = *0.0007 at E13.5). Notably, between E12.5 and E13.5, the mean proliferation rate within MRs decreases from 5.5 to 10.7-fold lower (*p<*0.0001) than the relatively constant rate of 22%±5% (*p>*0.09) in the adjacent ectoderm (Table 2). As Balinsky had concluded that as a pool, MRs grow negatively allometric with the embryo between E11 and E14 [4], we now asked whether this is true for individual MRs and on a day-to-day basis, and whether the relatively low proliferation rate in MRs would simply reflect such negatively allometric growth. We found that between E11.75 and E12.5, most MRs grow negatively allometric. However, MR2 then grows almost isometrically (2.5 versus 2.7 fold increase), and like MR4, grows positively allometric between E12.5 and E13.5 (Table 1). Therefore, proliferation rates of at least these MRs are too low to account entirely for their growth.

**Table 2:** Fold difference of ectodermal over mammary epithelial proliferation rate in wt E11.5–E13.5 mouse embryos.

	E11.5 (*n*)	E12.5 (*n*)	E13.5 (*n*)
**MR1**	3.5 (*2*)	4.5 (*4*)	5.5 (*3*)
**MR2**	1.3 (*2*)	5.9 (*4*)	24.8 (*3*)
**MR3**	5.6 (*4*)	4.0 (*4*)	6.5 (*3*)
**MR4**	2.5 (*4*)	8.9 (*4*)	8.1 (*3*)
**MR5**		4.2 (*4*)	8.5 (*3*)
**Mean**	**3.2**	**5.5**	**10.7 (7.1 w/o MR2)**

Numbers represent the mean ratios (Ectoderm/MR) as derived from the statistics on BrdU-incorporation represented in Fig. 2AC–AE. Abbreviations: wt: wild type; MR: mammary rudiment.

How much does proliferation contribute to wt MR growth? As the surface ectoderm of the trunk remains single-layered before E13.5, its proliferative activity must be approximately isometric with growth of the entire embryo. With *e.g.* the cells of MR2 having a 5.9-fold lower proliferation rate than the ectoderm (Table 2), proliferation may account for (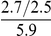)≈18% of the growth of this MR at E12.5, declining to (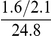)≈3% by E13.5. Similarly, the contribution of proliferation to MR growth may decline from ∼15%–44% to ∼3%–19% over 2 days in a rudiment-specific manner. Based on these rough estimates, we conclude that MRs grow neither entirely nor primarily by cell proliferation, and that cell proliferation contributes progressively less to MR growth between E11.5 and E13.5. The declining proliferation rates between E12.5 and E13.5 contrasting the steady or increased growth rate of MRs during this day (Fig. 1O), and the lower proliferation rate in MR2 and MR4 (Fig. 2AD, AE) despite their faster growth than MR1 and MR3 during this day (Fig. 1O), support this conclusion.

### Hypoplasia of Gli3^Xt-J/Xt-J^ MRs is not due to defective epithelial proliferation, and concurs with failure to downregulate mesenchymal proliferation

Interestingly, *Gli3^Xt-J/Xt-J^* MRs display hypoplasia despite their normal or increased proliferation rate at E12.5 (*p = *0.012 for MR2) and E13.5 (Fig. 2AD,AE). Given that Balinsky had suggested that the MRs grow due to cell aggregation from the adjacent ectoderm [5], we assessed whether the *Gli3^Xt-J/Xt-J^* ectoderm proliferates fast enough to provide cells for the MRs. Indeed it does, as shown by the equal to perhaps increased ectodermal proliferation rate in E11.75 *Gli3^Xt-J/Xt-J^* embryos compared to wt embryos (Fig. 2AC), and a rate similar to wt at E12.5 and E13.5 near all MRs (Fig. 2AD, AE), with the statistically significant difference near MR2 at E12.5 (*p = *0.025) perhaps being biologically insignificant.

Of interest, the ectoderm between the limbs tends to have a higher proliferation rate dorsally to the MRs, *i.e.* near MR2, MR3 and MR4, yet ventrally at the level of the forelimb (MR1) and hindlimb (MR5) in wt (Fig. 2AI). The higher proliferation rate of the ventral ectoderm seems to neutralize or switch towards a higher dorsal proliferation rate by E13.5. However, few of these differences are significant, giving no hint as to whether one side may contribute more cells to the MRs than the other. Furthermore, this tendency is not different in the *Gli3^Xt-J/Xt-J^* ectoderm.

Given the mesenchymal *Gli3* expression (Fig. 1P–V), we also assessed the mesenchymal proliferation rate. In E11.75 and E12.5 wt embryos, mesenchymal cells proliferate at a rate similar to ectodermal cells (Fig. 2AC,AD,AF,AG), with no biologically and statistically significant differences between dermal and ‘pre’mammary mesenchyme except near MR5 at E12.5 (*p = *0.014) (Fig. 2AG, AH). Between E12.5 and E13.5, a few layers of dermal mesenchyme directly adjacent to the MR condense and express the Androgen Receptor, marking their differentiation into mammary mesenchyme [24]. This mesenchyme reduces its proliferation rate less than the dermal mesenchyme by E13.5, resulting in significantly different rates between dermal and mammary mesenchyme near MR1, MR2 and MR3 (*p = *0.035, 0.038 and 0.012 respectively) (Fig. 2AH). The most prominent difference in *Gli3^Xt-J/Xt-J^* embryos is the significantly higher proliferation rate of dermal mesenchyme than in wt near MR2 (*p = *0.035) and MR4 (*p = *0.011) due to a failure to slow down proliferation between E12.5 and E13.5.

In conclusion, *Gli3^Xt-J/Xt-J^* MR2 and MR4 are hypoplastic despite having an initially higher proliferation rate than wt, particularly in MR2. Furthermore, the loss of *Gli3* changes cell proliferation in the mammary epithelium and mesenchyme, and in the adjacent ectoderm and dermal mesenchyme in a tissue-, location-, and time-specific manner.

### Hypoplasia of Gli3^Xt-J/Xt-J^ MRs 1, 2 and 4 is not caused by epithelial apoptosis

**Figure 3:**
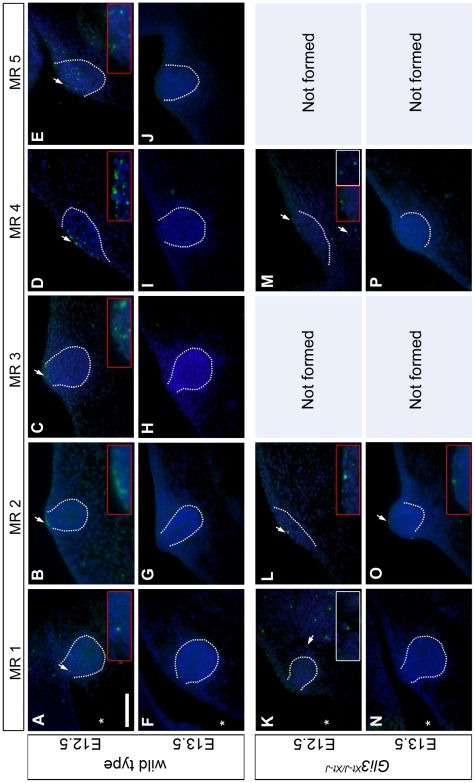
Apoptosis in MRs and their adjacent tissues is regulated by Gli3. **A–P:** Epifluorescence images of TUNEL-stained (green) and DAPI-counterstained (blue) central transversal sections through MRs, outlined by dashed white lines. Red insets: magnification of the apical area of the MR. White insets: magnification of a TUNEL^+ve^ region in the mammary mesenchyme. Left is ventral ectoderm, in some panels indicated with *. Scale-bar in A: 100 µm in A–P.

We then assessed whether the hypoplasia of *Gli3^Xt-J/Xt-J^* MRs could be due to apoptosis. TUNEL-assays show different apoptotic profiles among the five wt MR pairs, with MR5 showing a random distribution within its epithelium, while the other rudiments show mainly apoptotic cells at their apex or in their overlying periderm at E12.5 (Fig. 3A–E). By E13.5, apoptosis occurs in a few cells in the periderm overlying MR5, but not within wt MR1–4 (Fig. 3F–J). At both ages, the surrounding ectoderm and mesenchyme show virtually no apoptosis. Within the *Gli3^Xt-J/Xt-J^* MRs or their overlying periderm, apoptosis is strikingly less prominent to absent at both ages (Fig. 3K–P). Thus, the marked hypoplasia of the *Gli3^Xt-J/Xt-J^* MRs by E13.5 occurs despite the prevention of apoptosis in the epithelia. By contrast, while wt mammary and dermal mesenchyme undergo virtually no apoptosis, the dermal mesenchyme near E12.5 MR4 in *Gli3^Xt-J/Xt-J^* mutants undergoes a little. It remains possible that mesenchymal interactions with the mammary epithelium are consequently disturbed, compromising the growth of MR4.

### Influx of ectodermal cells from outside the ML contributes to MR formation and growth

**Figure 4:**
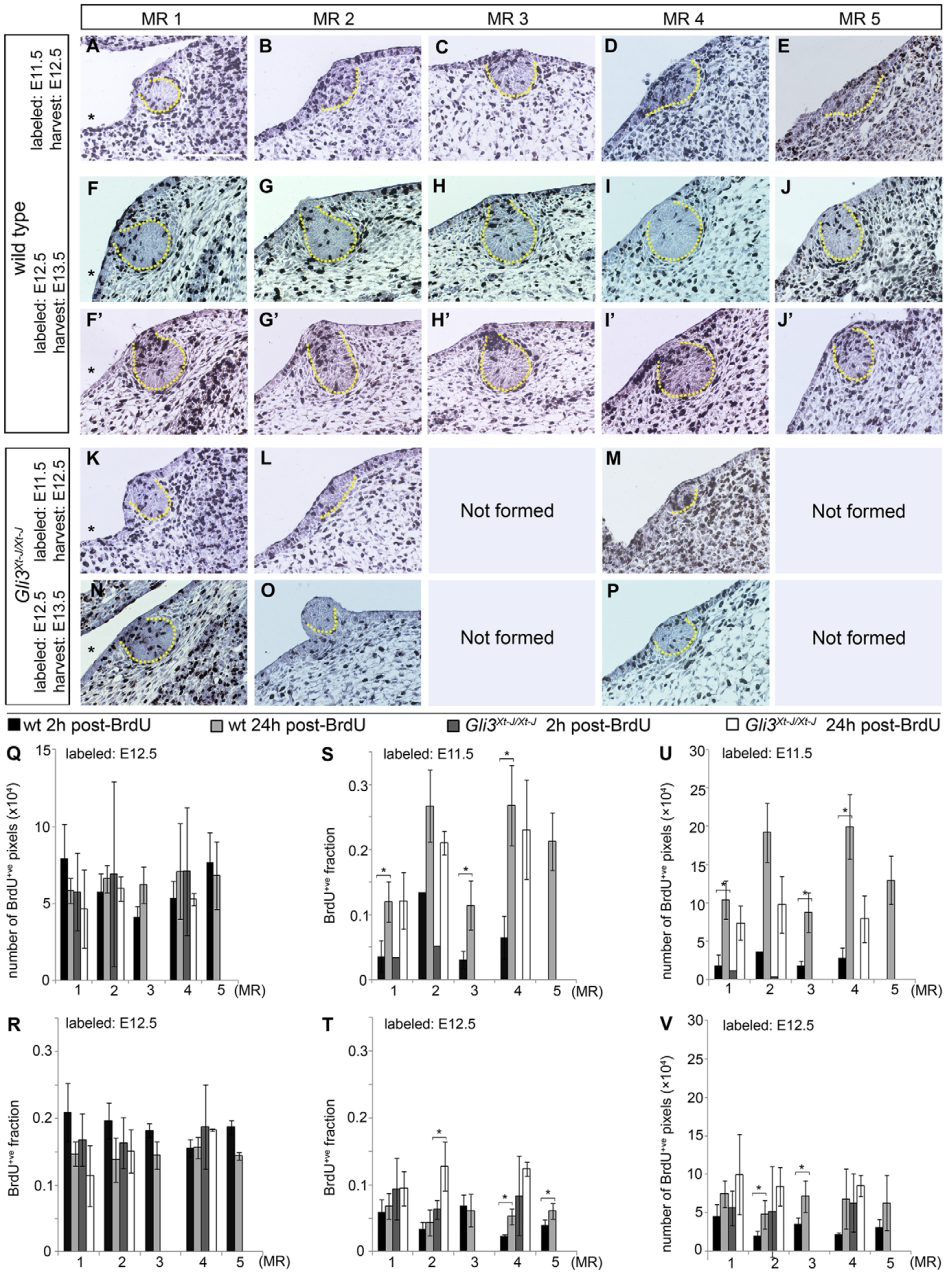
Ectodermal influx provides growth of all MRs and is perturbed for *Gli3^Xt-J/Xt-J^* MR2 and MR4. **A–P:** Immunohistochemical detection of BrdU-incorporation (black) at 24 hours post-labeling, with hematoxylin-counterstain (blue). Panels F–J and F′–J′ show a random versus primarily proximal distribution of labeled cells observed in 60% and 40% of wt MRs respectively. Dashed yellow lines outline the MR. Left is ventral ectoderm, in some panels indicated with *. Scale-bar in A: 100 µm in A–P. **Q–V:** Quantification of BrdU-labeling in entire MRs and adjacent tissues represented in A–P, by absolute number of BrdU^+ve^ pixels (Q) or BrdU^+ve^ fraction of all nuclear pixels (R) in all segmented tissues combined; or BrdU^+ve^ fraction of all nuclear pixels (S,T) or absolute number (U,V) of BrdU^+ve^ pixels in the MRs.

Next we asked whether the MRs recruit ectodermal cells for their growth. Unfortunately, the focal instability of flank cultures rendered time-lapse video-analysis of deposited dyes like DiI, or of conversion of the fluorescent substrate DDAOG, which visualizes expression of the mammary epithelial marker TOPGAL, fruitless for answering this question. However, the low and declining proliferation rate (4.5% to 3.3%) of cells in the MRs between E11.75 and E13.5 allowed us to BrdU-label embryos and track labeled cells 24 hours later (t = 24 h). A higher percentage of BrdU^+ve^ cells in the MRs at t = 24 h than at t = 2 h (as used in our proliferation assays) would be indicative of an influx of cells. In accordance with BrdU being taken up by cells quickly or otherwise degraded within 10–60 minutes [28], we did not observe a significant increase in absolute number or ratio of BrdU^+ve^ pixels in all tissues combined between t = 2 h and t = 24 h (Fig. 4Q,R). This provides proof of principle that postponed harvest did not prolong BrdU-labeling time in our experiments.

Given that all cells within the MRs are histologically and biochemically similar before E12.5 (MR1 and MR3) or E13.5 (MR2, MR4 and MR5), we may assume that at least until then all MR-cells are equal in cell cycle duration as well. Despite inheriting only half the amount of BrdU, both daughters of a symmetrically dividing BrdU^+ve^ cell each generate an equal number of BrdU^+ve^ pixels as their mother, because of saturation of the immuno-precipitate. Thus, if the MRs would grow by cell proliferation only, the ratio of BrdU^+ve^ pixels within the MRs at t = 24 h should fluctuate around the ratio at t = 2 h, and never reach twice that ratio, because unlabeled cells in M and G2 will divide prior to the cells labeled in S-phase.

We observed a 2 to 4-fold higher ratio of BrdU^+ve^ cells in MRs at t = 24 h compared to t = 2 h after labeling at E11.5 (p≤0.012) (Fig. 4S). The absolute number of BrdU^+ve^ pixels increased about 4-fold, varying per MR (p≤0.003) (Fig. 4U). If this increase were only due to mitosis of cells within the MRs, the MR volume should increase 4-fold as well, especially for MR2 and MR4 that hardly experience any cell apoptosis within them. Instead, these MRs only double their size in that day, indicating MRs use mechanisms other than cell proliferation alone to grow.

We infer that the increase of BrdU^+ve^ pixels is at least partly due to an extensive influx of cells. Given the ectodermal origin of mammary epithelium [3], the influxing cells must come from the more densely labeled ectoderm. A mathematical approach (Table 3) suggests that growth is approximately entirely due to such influx. Since the ML connecting the prospective MRs 2–4 in mouse embryos at around E11.5 is almost completely devoid of BrdU^+ve^ cells, any influx of cells from along the ML into those MRs would not be detected with our method. We can therefore deduce from the observed BrdU^+ve^ influx that most of the ectodermal cells recruited into the MRs by E12.5 were located outside the ML at E11.5.

**Table 3:** Estimated volume increase of mammary rudiments over 24 hours due to ectodermal influx.

1	2		3		4				5				6		7		8			9		10			11		12	
		Fold increase in absolute number of BrdU^+ve^ pixels within MR										÷	fraction BrdU^+ve^ in ectoderm @ t = 2 h	×	fraction BrdU^+ve^ in MR @ t = 2 h	=	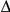 V_Exp_, _t = 24 h_ (fold incr.)	Fold increase in MR Volume (pixels)								=	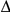 V_Obs_, _t = 24 h_ (fold incr.)	See foot-note
t = 0_ (labeling)_	MR	((	BrdU^+ve^ pixels _t = 24 h_	-	BrdU^+ve^ pixels _t = 2 h_	)		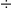		BrdU^+ve^ pixels _t = 2 h_	)							(	V_t = 24 h_		-	V_t = 2 h_	)	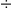	V_t = 2 h_			
E11.5	wt 1	((	103804	-	18340	)		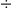		18340	)	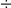	0.13	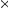	3.6E-2	=	1.29	(	220554		-	97777	)	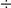	97777	=	1.26	1
E11.5	wt 3	((	87785	-	18659	)		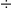		18659	)	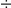	0.17	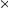	3.1 E-2	=	0.66	(	201646		-	131174	)	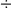	131174	=	0.54	1
E11.5	wt 4	((	199576	-	27841	)		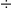		27841	)	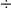	0.16	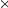	6.5 E-2	=	2.50	(	191118		-	95857	)	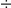	95857	=	0.99	1
E11.5	mt 1	((	73650	-	11775	)		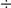		11775	)	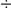	0.17	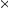	3.4 E-2	=	1.06	(	148758		-	74470	)	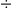	74470	=	1.00	
E11.5	mt 2	((	97784	-	3074	)		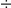		3074	)	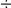	0.24	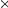	5.2 E-2	=	6.58	(	115751		-	14155	)	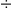	14155	=	7.18	
E12.5	wt 1	((	74388	-	44606	)		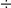		44606	)	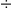	0.26	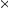	5.9 E-2	=	0.15	(	336218		-	220554	)	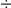	220554	=	0.52	2
E12.5	wt 2	((	47805	-	19050	)		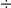		19050	)	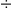	0.20	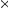	3.4 E-2	=	0.26	(	320787		-	154765	)	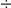	154765	=	1.07	2
E12.5	wt 3	((	71186	-	34501	)		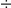		34501	)	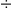	0.27	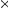	6.9 E-2	=	0.27	(	345423		-	201646	)	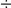	201646	=	0.71	2
E12.5	wt 4	((	68000	-	21156	)		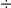		21156	)	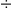	0.21	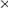	2.3 E-2	=	0.24	(	401653		-	191118	)	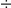	191118	=	1.10	2
E12.5	wt 5	((	62825	-	31123	)		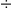		31123	)	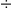	0.17	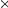	4.0 E-2	=	0.24	(	265012		-	163703	)	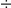	163703	=	0.62	2
E12.5	mt 1	((	99624	-	56019	)		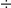		56019	)	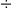	0.18	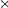	9.4 E-2	=	0.41	(	274814		-	148758	)	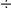	148758	=	0.85	3
E12.5	mt 2	((	71708	-	51318	)		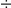		51318	)	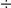	0.18	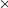	6.3 E-2	=	0.14	(	147332		-	115751	)	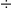	115751	=	0.27	3
E12.5	mt 4	((	85049	-	62714	)		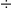		62714	)	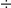	0.21	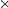	8.4 E-2	=	0.14	(	192489		-	131015	)	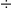	131015	=	0.47	3

Explanation of calculations: Unlabeled ectodermal cells — linearly correlating with pixel-number — have an equal chance of entering the MR as labeled ectodermal cells, their fraction correlating inversely to the labeled fraction of the ectoderm. Hence, the fold increase in BrdU^+ve^ pixels (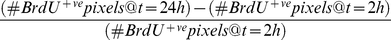), divided by the BrdU^+ve^ ectodermal fraction, yields the total number of influxing ectodermal pixels into MRs. This calculation is slightly overestimated, as the small fraction of BrdU^+ve^ cells within the MRs (column 7) is expected to undergo mitosis within 24 h, contributing to the increase in BrdU^+ve^ pixels at t = 24 h. The number of BrdU^+ve^ pixels (or cells) in column 5 constitutes the fraction of all cells in MR (*i.e* fraction of MR volume) in column 7. Thus, the fold increase in pixels (columns 3–6), multiplied by that fraction yields the expected volume increase, 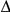
V_Exp_, if all additional pixels came from ectodermal influx. V_Obs_ is the observed MR volume, based on measurements of all pixels within the MR contours of consecutive sections throughout MRs. Abbreviation: MR: mammary rudiment.

1
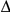
V_Obs_<
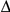
V_Exp_ due to proliferation and apoptosis in MR and apoptosis in overlying periderm.

2
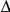
V_Obs_>
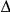
V_Exp_ due to hypertrophy of peripheral cells within all MRs.

3
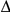
V_Obs_>
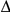
V_Exp_ due to hypertrophy.

**Figure 5:**
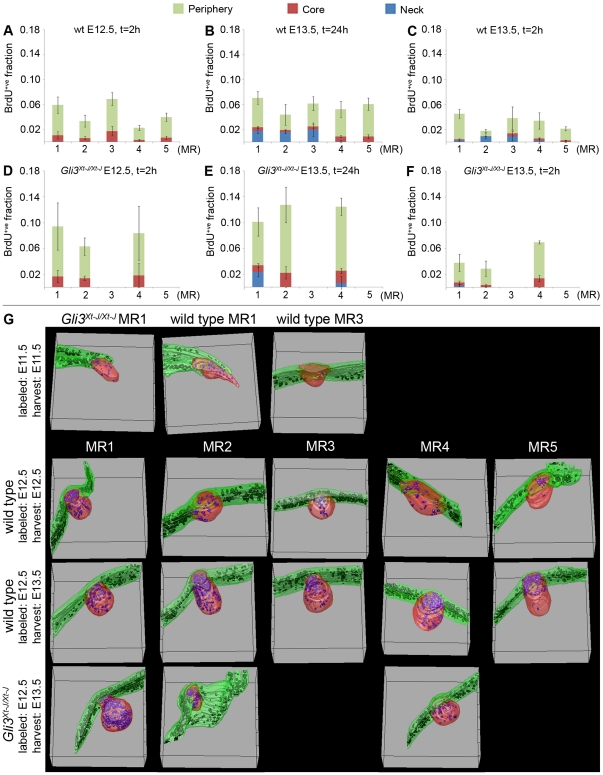
Unique compartmentalization of BrdU^+ve^ cells among individual MRs at E13.5. **A–F:** BrdU^+ve^ fraction of all nuclear pixels per MR (y-axis), subdivided proportionally by distribution over core, periphery, and neck if present. **G:** 3D-reconstruction of MRs based on serial sections stained for BrdU as in Figs. 2 and 5. Ectoderm/epidermis in green, with black BrdU^+ve^ nuclei; MR-epithelium in red , with blue BrdU^+ve^ nuclei.

Between E12.5 and E13.5, the ratio of labeled pixels in wt MR4 (*p = *0.004) and MR5 (*p = *0.03) and the absolute number of BrdU^+ve^ pixels in all wt rudiments still increases, but to a lesser extent (Fig. 4T,V). Subdivision of the MRs into a core, periphery and potential neck (*i.e.* in MR1-MR4 by E13.5), along with 3D-renderings of MRs show that BrdU^+ve^ cells labeled at E12.5 are predominantly located in the proximal region, *i.e.* neck if present, of the MRs by E13.5 (Figs. 4F′–J′, 5A,B,G). In the absence of such preferential proximal location at the time of labeling at E12.5 (Fig. 2G–P, 5G), these data strongly suggest that the increase of BrdU^+ve^ cells is caused by a continued ectodermal influx between E12.5 and E13.5.

### Ectodermal influx is decreased in Gli3^Xt-J/Xt-J^ MRs 2 and 4

In *Gli3^Xt-J/Xt-J^* MR1, the change in ratio, absolute number, and location of BrdU^+ve^ pixels is similar to that in wt between E11.5 and E13.5 (Figs. 2A,G,Q,T; 4A,F,K,N,S–V; 5A–F) consistent with a similar growth rate and morphogenesis as wt MR1 (Fig. 1O), and indicating no perturbation of migration. By contrast, the ratio of BrdU^+ve^ pixels in E12.5 *Gli3^Xt-J/Xt-J^* MR2 and MR4 labeled at E11.5 is almost as high as in wt (Fig. 4S), but their absolute increase in pixels over 24 hours is at least 2-fold less than in wt (Fig. 4U) despite an ectodermal labeling density similar to that in wt (Fig. 2AC,AD). This indicates a strongly reduced influx of ectodermal cells in *Gli3^Xt-J/Xt-J^* MR2 and MR4 (Fig. 4L,M), which accounts primarily for their hypoplasia seen at E12.5.

Between E12.5 and E13.5, the absolute number of BrdU^+ve^ pixels increases slightly in these MRs (Fig. 4T). However, the minimal growth of these MRs (Fig. 1O) and their failure to form a neck or proximally accumulate BrdU^+ve^ cells (Fig. 4O,P; 5G) indicate that ectodermal influx remains compromised, and may suggest that the increase in BrdU^+ve^ pixels would mostly be due to cell proliferation. Thus, continued compromised ectodermal influx complements the lack of hypertrophy in *Gli3^Xt-J/Xt-J^* MR2 and MR4 between E12.5 and E13.5, explaining the persistent hypoplasia of these MRs.

## Discussion

To date, knowledge about the cellular mechanisms of early mammary rudiment (MR) growth in the mouse embryo was based largely on assumptions and controversial extrapolations. Therefore, we here explored the nature of these mechanisms. In view of the initially daily switching morphogenetic stages, and each of the five MR pairs having a differential requirement for Gli3 for their induction and development, we analyzed each of the MR pairs individually, at discrete days, in wt and *Gli3^Xt-J/Xt-J^* (null) mouse embryos.

### Relative contributions of ectodermal influx, proliferation, apoptosis, and differentiation into hypertrophic columnar cells, to mammary rudiment growth

In line with Balinsky's data [4], we identified a lower proliferation rate in the mammary epithelium compared to its surrounding ectoderm. While Balinsky had pooled all - or an unknown subset of - MRs of E11–E14.5 embryos for statistical analysis, we show here that significant differences in proliferative activity exist within each MR at E11.5, E12.5 and E13.5. At any of those days, the MRs also differ significantly from each other in proliferative activity. We provide three independent lines of evidence that the proliferative activity is too low for MRs to grow entirely, or even primarily, by cell proliferation between E11.5 and E13.5:

1. the proliferation rate declines, contrary to the constant or increasing growth rate of the MRs,
2. the average 5.5 to 10.7-fold lower proliferation rate of cells within MRs compared to the ectoderm is inconsistent with the only slightly negative or even positive allometric growth of the MRs with the embryo between E11.5 and E13.5, and
3. the fold increase of BrdU^+ve^ cells at 24 h compared to 2 h post-labeling at E11.5 is much greater than the fold increase in MR volume in that time period.

We estimate that cell proliferation contributes initially ∼15%–44% to MR growth, declining to ∼3%–19% between E12.5 and E13.5, all in a rudiment-specific manner. Cell proliferation may take on a more important role in MR growth between E14.5 and E15.5 [29].

We demonstrate that MRs grow initially by an influx of ectodermal cells. This influx is large during the first day, accounting for all growth that is not mediated by cell proliferation within the MRs. The influx decreases such that we estimate it to contribute 22%–39% of MR growth between E12.5 and E13.5, again in a rudiment-specific manner (see Table 3,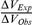). Ectodermal influx may continue for one more day, as indicated by our previously published observation of similarly proximally located labeled cells in E14.5 MRs in cultured flanks, labeled a day earlier with [^3^H]-thymidine [30].

Our results indicate that most of these influxing cells were located outside the ML between E11.5 and E12.5, prior to their incorporation in the emerging MRs in the course of 24 hours. Our data may either support Balinsky's suggestion of centripetal aggregation of ectodermal cells toward the MR positions [5], or the possibility that within those 24 hours, cells first migrate towards the ML, and subsequently migrate along this line towards the prospective MRs. The latter possibility may be supported by the published observation that DiI-labeled ectoderm posterior to the forelimb bud of a cultured E10 mouse embryonic flank expanded in ventral-posterior direction over 72 hours [31]. However, since no time-lapse video-analysis was performed during those 72 hours, it is unclear whether this expansion resulted from cell proliferation and distortion of flanks during such long-term culture or from ectodermal cell migration in dorso-ventral direction and antero-posterior direction. If indeed the latter, then our results would indicate that migration toward the ML and along the ML would occur simultaneously in the mouse embryo. This contrasts what happens in the rabbit embryo, where formation of the mammary ridge seems to be temporally separated from cell migration along the mammary ridge towards the prospective MRs [6], [7].

Based on our finding of the involvement of somitic Gli3 and FGF10 in ML and MR3 formation, we previously suggested that in between the forelimb and hindlimb, ectodermal cells may be pulled along in dorso-ventral direction with the ventrally elongating somites to form the ML [9]. Along that line of thinking, our current finding of a higher proliferative activity - albeit it not statistically significant – dorsally than ventrally to MR2, MR3 and MR4, but not MR1 and MR5, is of interest: It may suggest that the dorsal ectoderm in the interlimb region is generating more cells to be pulled ventrally toward the mammary line, while in the area of MR1 and MR5 another mechanism – and directionality - of ectodermal recruitment may be active. While this scenario does not necessarily exclude additional recruitment of ventral ectoderm, it remains of interest, yet experimentally challenging, to determine the exact extent and directionality of ectodermal influx into each of the MRs.

How do the MRs maintain or even increase their growth rate between E12.5 and E13.5 if both cell proliferative activity within the MRs and ectodermal influx are declining? We show here a not previously considered mechanism of MR growth, namely by hypertrophy of their peripheral cells between E12.5 and E13.5. We estimated that hypertrophy could explain perhaps a 1.7 fold increase in MR-volume, which would equal 80%–100% of growth in that day in a rudiment-specific manner. All estimations together (i.e ∼3%–19% proliferation +22%–39% cell migration +80%–100% hypertrophy) add up to more than 100% of the growth between E12.5 and E13.5, which may be partly explained by the fact that they are estimations; partly by the possibility of a differential contribution to the influx by the peridermal and germinal layer of the ectoderm which have seemingly different proliferation rates; and partly by the need of growth-promoting mechanisms to compensate for the loss of cells by apoptosis occurring to different extents among the MRs within themselves and/or in their covering periderm at around E12.5.

**Figure 6:**
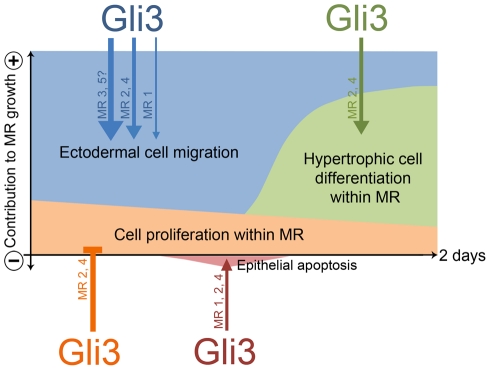
A model of the role of Gli3 in regulating mammary rudiment induction and early growth. See discussion for explanation. Thickness of arrows relates to the relative extent of involvement of Gli3 in a process.

Figure 6 shows a model of the contribution of all four abovementioned mechanisms to MR growth. While the sequence of mechanisms seems similar for all MRs, these mechanisms occur asynchronously among all MRs, and contribute to different extents to each of the MRs at any point of time. These differences may simply reflect the asynchronous emergence of the MRs [1], [18]. Alternatively, they may reflect molecular differences among the MRs, as demonstrated by the variation in phenotype among MRs in *Gli3^Xt-J/Xt-J^* embryos.

### Rudiment-specific roles of Gli3 in cellular processes governing mammary rudiment growth

We identified that the five mammary rudiments (MRs) in the mouse embryo display differential responses to the absence of *Gli3*: While MR3 and MR5 are not induced at all, MR1, MR2 and MR4 are about ¼ day delayed in the onset of their formation. Once induced, MR1 follows a normal growth rate and morphogenesis, but MR2 and MR4 experience a slow growth until at least E13.5. Moreover, MR2 protrudes outwardly instead of invaginating into the underlying dermis. This protrusion remains unexplained mechanistically, but may be related to the higher proliferation rate in the dermal and mammary mesenchyme at E13.5 and the failure of ectoderm covering the MR to undergo apoptosis. We could attribute the stunted growth of both rudiments largely to a defect in ectodermal influx between E11.5 and E12.5, complemented by a failure of peripheral cells to differentiate into hypertrophic, columnar cells between E12.5 and E13.5.

How would Gli3 regulate these cell fate decisions? As described below, Gli3 has been shown to play a role in proliferation, survival, migration and hypertrophy of a variety of cell types. Depending on cell type, it executes its role either as a full length transcriptional activator (Gli3A) or a truncated repressor (Gli3R) form. In general, high levels of Hedgehog (Hh) signaling maintain Gli3 as Gli3A, leading to transcription of the Hh receptor *Ptc1* and the transcriptional activator *Gli1* as a feed forward mechanism of Hh signaling. In the absence of Hh signaling, Gli3 becomes a transcriptional repressor (Gli3R) [32].

Hh signaling and cell-autonomously acting GliA family members have migration promoting effects on *e.g.* enteric nerves, pancreatic stellar cells and endothelial cells [33], [34], [35]. However, it is very unlikely that Gli3 regulates ectodermal cell migration as GliA, because Gli3 is not expressed in the ectoderm and presumptive mammary epithelium at around E11.5, and although it is expressed in the MRs at E12.5 and E13.5, it most likely functions as Gli3R as suggested by the absence of expression of *Ptc1* and *Gli1* [9], [19], [36]. Instead, the migration defect during formation and early growth of MR2 and MR4 is likely non cell-autonomous and a consequence of perturbed mesenchymal-ectodermal interactions due to a lack of Gli3 function in the somites or other mesenchymal tissue [9]. We speculate this would be a GliR function, based on increased *Gli1* expression in the mammary mesenchyme of *Gli3^Xt-J/Xt-J^* MR2 at E13.5 (Fig. S1; other MRs not investigated) and of MR1 and MR4 at E14.5 [9], the lack of requirement for Shh for induction of MRs [37], [38], and similar to the requirement for Gli3R and the repression of Hh-signaling in formation of MR3 and MR5 [19]. Along the same line, we now assume that the failure to form MR3 and MR5 is due to a more severe ectodermal migration defect than for MR2 and MR4.

Similarly, our observed reduction of mammary epithelial cell proliferation in MR2 and MR4, and apoptosis in MR2-MR5 in the presence of Gli3 would be consistent with high Gli3R activity repressing cell cycle progression and promoting cell apoptosis cell-autonomously in neural progenitor cells, as reviewed [39].

During skeletogenesis, Gli3R represses differentiation of distal chondrocytes into columnar and subsequently hypertrophic chondrocytes [40]. If Gli3 similarly regulates the hypertrophy of cells within MRs, the failure of peripheral cells in *Gli3^Xt-J/Xt-J^* MR2 and MR4 to become larger columnar cells by E13.5, would suggest Gli3 normally acts as Gli3A in these cells. Given the unlikeliness of Gli3 being present as Gli3A in these cells, as argued above, differentiation of the peripheral cells into larger columnar cells in MR2 and MR4 perhaps depends non-cell-autonomously on Gli3-mediated communicative signals emitted by the mesenchyme.

### Summary and implications

We have answered the long-standing question of which cellular mechanisms drive the onset of formation and early growth of MRs in the mouse. Gli3 plays a role in all processes involved, *i.e.* proliferation, migration and differentiation into hypertrophic columnar cells, as well as apoptosis, apparently regulating the choice between the different fates a cell can have. As Gli3 remains expressed in the adult mammary gland [19], it may then perhaps likewise regulate the balance between normal homeostasis and tumor growth, as supported by the recent identification of Gli3 in a primary breast tumor expression dataset [41], and similar to what has recently been reviewed for several other genes [42], [43]. Of note, despite the similarities among MRs regarding the cellular mechanisms regulating their early growth, the function of *Gli3* in these mechanisms differs among the MRs, indicating MRs are not mere copies of the same structure but distinct entities, as we have suggested previously [2]. Such differential molecular involvement among the various mammary glands may provide a beginning to an explanation how differences in numbers and positions of mammary glands are created among mammals, and simultaneously raise the question whether all five pairs of murine mammary glands can be considered equally appropriate models for the human breast.

## Supporting Information

Figure S1 ***Gli1*** **mRNA expression is upregulated in** ***Gli3^Xt-J/Xt-J^*** **mammary mesenchyme of MR2.** Bright-field images of **A:** E13.5 wt and **B:** *Gli3^Xt-J/Xt-J^* MR2. **A′,B′:** corresponding dark-field images with the radio-active *in situ* hybridization signal of a *Gli1* mRNA probe in white. MRs are outlined with dashed black lines. White arrow points at the high hybridization signal in the mammary mesenchyme directly underlying the *Gli3^Xt-J/Xt-J^* MR2.
(TIF)
