# Supplementary figures and images for: Ectodermal Influx and Cell Hypertrophy Provide Early Growth for All Murine Mammary Rudiments, and Are Differentially Regulated among Them by Gli3
Source: PLoS One. 2011 Oct 27;6(10):e26242. doi: 10.1371/journal.pone.0026242 (PMC3203106; doi:10.1371/journal.pone.0026242)

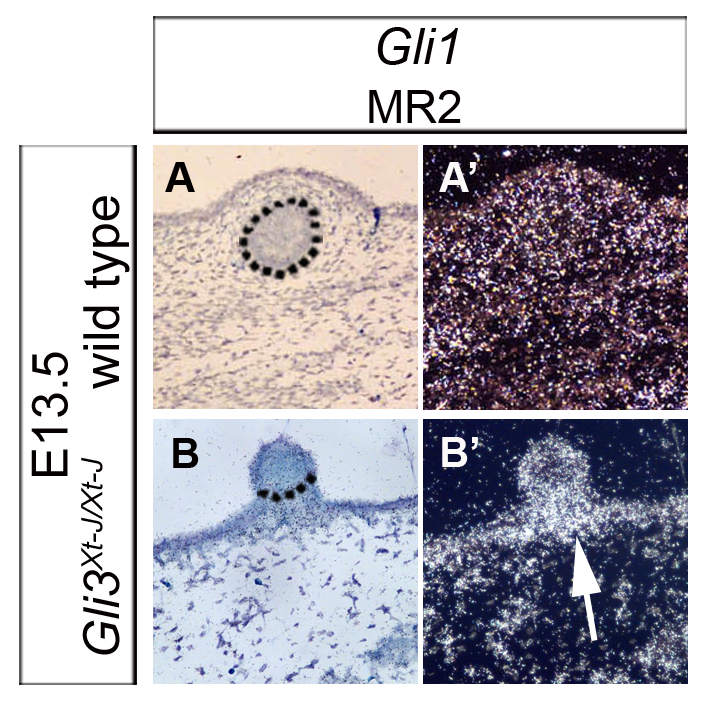

Supplement: Figure S1 — Gli1 mRNA expression is upregulated in Gli3Xt-J/Xt-J mammary mesenchyme of MR2. Bright-field images of A: E13.5 wt and B: Gli3Xt-J/Xt-J MR2. A′,B′: corresponding dark-field images with the radio-active in situ hybridization signal of a Gli1 mRNA probe in white. MRs are outlined with dashed black lines. White arrow points at the high hybridization signal in the mammary mesenchyme directly underlying the Gli3Xt-J/Xt-J MR2. (TIF) [file pone.0026242.s001.tif]
